# Dengue as a Disease Threatening Global Health: A Narrative Review Focusing on Latin America and Brazil

**DOI:** 10.3390/tropicalmed8050241

**Published:** 2023-04-23

**Authors:** Carlos Letacio Silveira Lessa, Katharine Valéria Saraiva Hodel, Marilda de Souza Gonçalves, Bruna Aparecida Souza Machado

**Affiliations:** 1 Postgraduate Program in Industrial Management and Technology, SENAI CIMATEC University Center, Salvador 41650-010, Brazil; 2 Gonçalo Moniz Institute, Oswaldo Cruz Foundation (IGM-FIOCRUZ/BA), Salvador 40296-710, Brazil; 3 SENAI Institute of Innovation (ISI) in Health Advanced Systems (CIMATEC ISI SAS), SENAI CIMATEC University Center, Salvador 41650-010, Brazil; 4 Anemia Research Laboratory, Department of Clinical and Toxicological Analysis, Faculty of Pharmacy, Federal University of Bahia, Salvador 40170-115, Brazil

**Keywords:** dengue, tropical disease, public health, Brazil, tropical areas

## Abstract

Arboviruses constitute the largest known group of viruses. These viruses are the etiological agents of pathologies known as arboviruses, with dengue being one of the most prevalent. Dengue has resulted in important socioeconomic burdens placed on different countries around the world, including those in Latin America, especially Brazil. Thus, this work intends to carry out a narrative-based review of the literature, conducted using a study of the secondary data developed through a survey of scientific literature databases, and to present the situation of dengue, particularly its distribution in these localities. Our findings from the literature demonstrate the difficulties that managers face in controlling the spread of and planning a response against dengue, pointing to the high cost of the disease for public coffers, rendering the resources that are already limited even scarcer. This can be associated with the different factors that affect the spread of the disease, including ecological, environmental, and social factors. Thus, in order to combat the disease, it is expected that targeted and properly coordinated public policies need to be adopted not only in specific localities, but also globally.

## 1. Introduction

Arboviruses are the largest known group of viruses. These viruses are maintained in nature through biological transmission between susceptible vertebrate hosts and hematophagous arthropods, or through arthropod-to-arthropod transmission via the transovarial route [1]. Diseases such as Zika, chikungunya, yellow fever, and especially dengue, are the most epidemiologically and clinically relevant arboviral diseases [2]. Dengue is a disease caused by an arbovirus of the genus *Flavivirus* and belonging to the family Flaviviridae, transmitted by the bite of *Aedes aegypti* or *Aedes albopictus* [3]. The dengue virus is composed of a single strand of ribonucleic acid (RNA) that has an icosahedral capsid protein coat. Four main viral serotypes (DENV-1, DENV-2, DENV-3, and DENV-4) cocirculate among humans, and these are genetically related, but antigenically distinct [4]. In 2013, data regarding a fifth serotype (DENV-5) were published based on an identification in Malaysia, Asia, which alerted the scientific community to new epidemiological and clinical aspects of the disease [5,6]. Among the different dengue virus serotypes, DENV-1 stands out as the most virulent, since it has the potential to cause major epidemics in a short period of time.

Dengue is distributed along the equator, and until the mid-1990s, the Southeast Asian region was the main territory afflicted by epidemics of the disease [7]. From this period onward, the countries of the Americas, which present environmental characteristics favorable for the development of the disease, started to account for more than half of the registered cases in the world, with a significant number of notifications of cases in Brazil in 1998 [8]. The epidemiological and statistical data shared through epidemiological surveillance efforts have recorded the incidence of dengue worldwide, pointing to its presence on different continents, even at different magnitudes [9]. Over the past three centuries, isolated epidemics have occurred on several continents, such as the Americas, Asia, and Europe, and in several countries, such as Australia, with an exacerbation in Southeast Asian countries. There has been an increase in the intensity of the disease in the form of epidemics or endemics, which have caused thousands of deaths, predominantly in children [10,11,12]. One of the factors associated with this characteristic is the fact that dengue is a disease that manifests itself mainly in periods of heavy rainfall and, depending on the social determinants, can cause serious harm to the population; thus, public health actions become essential for its control [13].

Brazil is one of the countries in the world where this behavior is most demonstrated, since data point to precarious housing and income conditions as factors that significantly contribute to increases in the disease; specifically, a lack of robust sanitation services is directly associated with the emergence of dengue [14]. Additionally, it is noted that, even in more favorable socioeconomic conditions, the absence of environmental awareness associated with the frequent habits in the cities of the country, such as the cultivation of aquatic plants and improper disposal of recyclable waste, may present risks for the emergence of the disease [15]. Therefore, from epidemiological studies, it is noted that different risk factors that can lead to the susceptibility to and transmission of dengue are related to the social determinants of health. Internal migration, violence, poverty, disorderly growth, illegal deforestation, and deficiencies in environmental management may indirectly promote the proliferation and spread of dengue [16]. Epidemiological studies not only provide an overview of dengue, but are also relevant in specific studies on genotypic variations that map viral changes that can trigger new epidemics, especially in the most severe manifestation of the disease (dengue hemorrhagic fever), resulting in an increase in the number of deaths [17].

In this context, epidemiological studies are capable of demonstrating the social determinants of health and are important from a public health point of view. Studies with these characteristics have an impact on the development and maintenance of a certain disease in a population or territory, since their approach considers not only technological actions in health, but also the existence of social inequalities, social relationships, social capital, and other issues predominant in their development [18]. Thus, as long as there is no change in these living conditions and geographical spaces, despite all the knowledge acquired over time, there will always be difficulties and possible failures in controlling dengue [17,19]. In view of the above, the aim of this review is to present the situation of dengue and its distribution around the world, specifically in the Americas and Brazil, by mainly considering the epidemiological aspects of the disease and the economic burden it imposes. The article also seeks to update readers on the difficulties related to the control of the disease and the planning of the measures to achieve this, as well as the main perspectives on this issue.

## 2. Disease Overview

### 2.1. Transmission Mode and Clinical Manifestations

**Figure 1:**
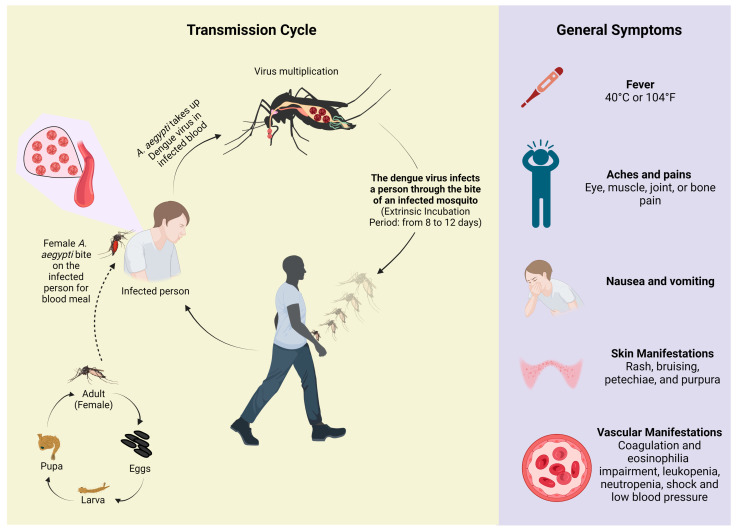
Overview of the dengue transmission cycle and the main symptoms related to the disease. The transmission system of the dengue virus begins when the mosquito bites an infected person; the virus multiplies in the gut of the insect and passes into other organs, finally reaching the salivary glands, from where it must exit through the bite into the bloodstream of another person not yet infected. Created in BioRender.com (accessed on 14 March 2023).

Dengue is the most prominent mosquito-borne virus, resulting in *Ae. aegypti* being an important target for preventing viral spread. Thus, dengue cannot be spread directly from person to person [20]. Dengue virus strains circulate through *Ae. aegypti* and humans (Figure 1) [21]. It is highlighted that the dengue virus is transmitted by female mosquitoes, mainly of the *Ae. aegypti* species and, to a lesser extent, of the *Ae. albopictus* species [22]. Clinical manifestations of dengue are diverse, ranging from mild to more severe symptoms, since dengue virus infection causes a syndrome that is initially benign and mild and rarely fatal. However, it may evolve into a hemorrhagic form that is more life-threatening due to increased vascular permeability, leading to systemic shock and abrupt multiple organ failure [23]. Many of these complications are a consequence of the pathophysiology of the disease, since the dengue virus has the ability to replicate in blood cells, such as in macrophages, reaching the bone marrow, and in certain situations, may compromise the production of platelets [24]. In addition, during their viral reproduction, they are able to produce substances that attack and damage the walls of blood vessels, and this may lead to a loss of plasma [25]. This severe form is a complication of dengue that can also be called severe dengue or dengue hemorrhagic fever, and is associated with altered blood coagulation, leading to capillary permeability. Further, if not diagnosed and treated correctly, it can result in patient mortality [26].

Clinical manifestations are usually more severe in individuals reinfected with different serotypes of DENV, increasing the risk of mortality. Primary infection is capable of conferring protective immunity (e.g., IgG production), while reinfection with a different serotype may be contrarily pathogenic (antibody-dependent enhancement hypothesis) [27]. This trend becomes an even more worrisome factor when it comes to the contexts imposed by globalization, where infected individuals move from endemic regions to places where dengue presents controlled epidemiological data or where a different serotype is circulating. Thus, the clinical and molecular surveillance of dengue is needed in both endemic and nonendemic regions to support public health measures to control the disease [28].

### 2.2. Management Aspects

Despite the existence of recommendations and guidelines on the care of individuals with dengue by health authorities, it is noted that the diagnosis of the disease in both its factory stage and more critical phases is a challenge, since the clinical symptoms and laboratory features may overlap or are similar to other diseases [29]. This condition occurs especially with diseases of viral-infectious nature, such as other arboviruses [30,31] and COVID-19 [32], which can lead to the situation of under-reporting dengue cases, a fact that can directly affect different areas of public health. It should be noted that individual case management is beyond the scope of this review, since it requires a targeted and specific analysis.

### 2.3. Disease Control

The treatment of dengue is considered a major challenge, since dengue virus-specific antivirals are not available, and the approved vaccines have recently received the release for use or have important limitations [33]. The first vaccine approved for use was the Dengvaxia vaccine (also known as CYD-TDV), developed by the French company Sanofi Pasteur [34]. Dengvaxia is a vaccine based on live-attenuated and tetravalent technology, whereby each of the four dengue virus serotypes was obtained separately by recombinant DNA technology and then combined with the attenuated yellow fever vaccine virus [23]. In general, it has been observed that the effectiveness of Dengvaxia depends on particular conditions, such as the virus serotype, whether or not there has been a previous infection (serological status), and the age of the individual receiving the vaccine [3,35,36]. Such constraints mean that the recommendation for the use of the vaccine was not extended to a wide population, limiting its use.

In light of this, different efforts have been made to obtain better-performing vaccines. In 2022, the vaccine TAK-003 (trade name QDENGA^®^) was approved by the Indonesia National Agency for Drug and Food Control and the European health authority, and subsequently, the same decision was given by the UK’s Medicines and Healthcare Products Regulatory Agency and the National Health Surveillance Agency in Brazil [37,38,39]. The TAK-003 vaccine is based on live-attenuated technology with a tetravalent property, since it has four DENV strains based on a DENV-2 backbone [34]. After 19 clinical trials (considering phases I, II, and III) conducted especially in dengue-endemic regions (such as Latin American and Asian countries), the vaccine demonstrated a safe and effective profile in individuals between 4 and 60 years of age to prevent dengue (preventing 80.2% of symptomatic dengue cases 12 months after vaccination), including the need for hospitalization (preventing 90.4% of hospitalizations 18 months after vaccination) [40,41]. The approval of the TAK-003 vaccine is greeted with great enthusiasm, as it has characteristics that go beyond the limitations of the previously approved dengue vaccine (such as previous serostatus), and may contribute significantly to the control of the disease.

Given the therapeutic limitations presented by the vaccine and the absence of specific drugs, vector control programs have gained importance in the fight against dengue [42]. The control of the viral vector goes through different initiatives, such as chemical and biological control strategies, sanitary legislation, environmental management, and, above all, the engagement of the population within this context. Lima et al. [43] demonstrated that integrated vector control strategies are the most successful and that when there is community participation, the results are even more effective. One of the ways to achieve the population’s engagement is through social mobilization by groups, neighborhoods, or communities, which involves recycling actions, distribution of educational material, and initiatives for the general cleaning of environments [44]. Such initiatives are entirely important for vector control.

Furthermore, health authorities have employed the use of insecticides (chemical control) [45], as well as providing biological control by means of predatory or genetically modified mosquitoes released in endemic locations [46]. Due to advances in molecular biology regarding gene editing techniques, the use of genetically engineered mosquitoes has gained popularity in recent years. The use of this approach usually has two general goals: population suppression or population modification [47]. The use of genetically engineered mosquitoes has been able to decrease the spread of the dengue virus in some localities in different parts of the world, impacting the reduction in the number of symptomatic cases and hospitalizations caused by dengue [48]. However, the use of this approach still fosters discussions by the scientific and local communities regarding ethical aspects (consent of individuals and/or the community to be exposed to genetically modified organisms), as well as a critical analysis between the possible risks and benefits in the environmental and public health spheres [49].

Traditionally, chemical mosquito control has been necessary when trying to control dengue transmission [45]. According to the WHO, during the period of 2010–2019, the annual global amount of insecticides used for disease vector control was more than 3000 tons [50]. The amount of insecticides employed for dengue control is only just less than for malaria control; however, it is important to note that this amount may be underestimated, since the strategies for vector control are usually implemented by municipal or district authorities who do not always share data on insecticide use with central agencies under the WHO [50]. It is noted that during outbreak periods, the spraying of insecticides in these locations is a measure widely adopted by health authorities. However, the occurrence of mosquitoes that have developed resistance to insecticides has been reported due to the presence of mutations that confer resistance by different mechanisms, such as reduced penetration of the insecticide and changes in mosquito behavior, compromising the efficiency of this strategy [33]. Countries in Latin America and South Asia have already reported the presence of resistant mosquitoes in their territory, and in these locations, this approach has raised questions [33].

Due to the limitations presented by the aforementioned strategies, preventive treatment or treatment during a viral infection, as well as the control of the mosquito vector itself, different studies have been conducted to assess the impacts of these tools in isolation or in association [51,52].

## 3. Dengue in the World

### 3.1. Epidemiologic Overview

Dengue is considered to be a serious disease that causes severe and harmful problems to the public health of the world population by the WHO, since the number of cases and associated deaths have increased dramatically over the last few decades [53]. Projections indicate that more than 6 billion people in 2080 will be at risk for dengue, more than double the cases that occurred in 2015 [54]. Dengue is believed to affect at least 4 billion people worldwide, equivalent to 50% of the world population, with a forecast of approximately 400 million infections per year, being symptomatic from 50 to 100 million [55]. These numbers mean that, among arboviruses, dengue is the most prevalent worldwide and is present mainly in tropical and subtropical countries, as well as in Southeast Asia, the Pacific, and the Americas, jeopardizing the health of more than 2.5 billion people (Table 1) [56].

The resurgence of dengue has been associated with a number of factors, such as geographic dispersion, increased global travel, population growth, and disorderly urbanization, which have all led to an imbalance in the environment in which we live, including an increase in the severity and lethality of the disease [59]. Thus, the prevalence and incidence of dengue fever worldwide have been associated with, among other factors, issues linked to globalization [60]. Infected individuals undertaking international travel can carry the virus from endemic regions to nonendemic locations, thus facilitating the wide spreading of dengue [61]. Another factor that deserves mention is climate change, a problem that has had critical public health outcomes, including its influence on dengue epidemiological rates [62]. It is believed that climate factors are able to influence dengue in an ecological manner, since they can modify the growth dynamics of the mosquito, as well as viral replication and its interaction with humans [63]. Additionally, it is expected that, with changes in temperature, nonendemic regions will have a larger contingent of mosquitoes capable of dengue virus transmission, thus expanding its distribution [64]. In association with this, the diagnosis of dengue is often neglected in these locations due to its mild symptoms and being undifferentiated from other viruses, making it difficult to treat and take measures against the transmission of the disease [65]. Misdiagnosis can cause infected individuals to become the “index case” for a given nonendemic region [9].

### 3.2. Economic Burden

The epidemiological contextualization of dengue also has an important socioeconomic implication, since it is estimated that the total costs worldwide, considering the direct (direct medical cost) and indirect (costs associated with time lost because of illness or care) expenses, amount to USD 8.9 billion annually [66]. This cost is higher than that found for other diseases, such as what was reported in the study by Hung et al. [67], which demonstrated that the laboratory cost for patients admitted to an intensive care unit in Vietnam is higher for those with dengue than for those with sepsis or tetanus. Additionally, the costs per dengue case are higher in countries with greater financial power than those with economic limitations. For example, Shepard et al. [66] demonstrated that the cost per case of dengue fever in the African region was around USD 56.00, while in countries such as the United States and Australia, this amount can reach USD 1146.00. Furthermore, the authors indicate that the overall mean values per dengue case also vary according to the type of treatment received by the individual, with the outpatient cases costing USD 51.16; however, when there is a need for hospitalization, this value can reach USD 70.10 [66]. These data indicate that the costs of dengue vary across different parts of the world (Table 2).

The data reported in Table 2 point to the complexity encountered when dealing with the economic burden of dengue, since between countries in the same region, these values can vary significantly. An example of this trend is when we compare Cambodia and Singapore, two Asian countries with a per capita cost of the illness having a difference greater than 1000% [69]. Typically, dengue-related costs are linked to patient care in healthcare facilities and the strategies used to control the vector that transmits the virus; in this case, the mosquitos *Ae. aegypti* and/or *Ae*. *albopictus*. However, it is important to emphasize that the economic analysis of dengue should involve the consequences linked to the disease, such as the inclusion of indexes such as health-related quality of life, going beyond hospitalization, mortality, and morbidity [77]. Furthermore, it is expected that the costs of dengue will increase in the coming years due to climate change, which may favor the spread of the vector and the neglected treatment by the health authorities against this disease [13]. Thus, the economic burden of the disease is expected to increase globally.

## 4. Dengue in the Americas

**Figure 2:**
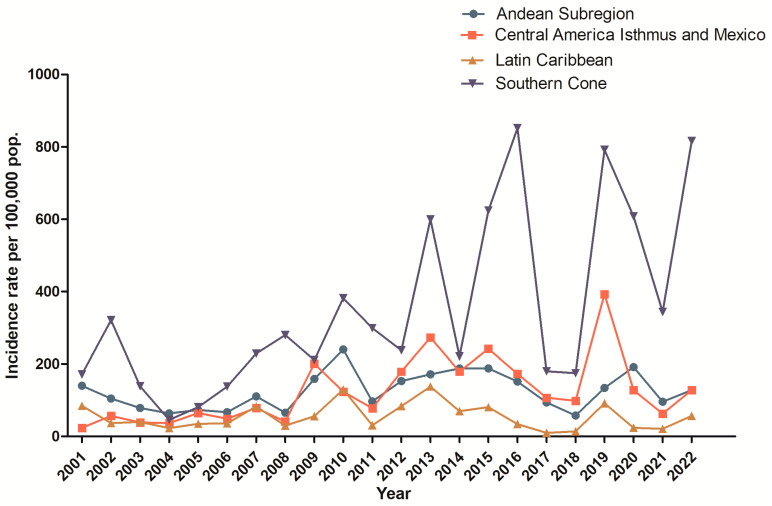
Dengue incidence rate per 100,000 inhabitants in the different regions of Latin America: Andean (countries: Bolivia, Colombia, Ecuador, Peru, and Venezuela); Central America Isthmus and Mexico (countries: Panama, Costa Rica, Nicaragua, Honduras, El Salvador, Guatemala, Belize, and Mexico); Latin Caribbean (countries: Cuba, Dominican Republic, and Puerto Rico); and Southern Cone (countries: Argentina, Brazil, Chile Paraguay, and Uruguay). Source from data: PAHO [80].

Latin America comprises the largest part of the American territory, since it includes countries from South America, Central America, and Mexico (North America), presenting a great diversity of geographical, political, and social factors. These particularities of each region are also reflected in dengue incidences, which vary between the endemic and nonendemic regions (Figure 2). Since 2010, the Americas have been facing the growing emergence and re-emergence of dengue and other viruses transmitted by the *Aedes* mosquitoes, raising global concerns about their public health consequences, as well as the feasibility of their prevention and control [78]. One of the remarkable characteristics in all the countries of the Americas, with the exception of the United States and Canada, is related to their state of social inequality, low income, lack of basic sanitation, and lack of employment and housing, which makes the determinants of health extremely relevant to the health conditions of their populations [79].

Additionally, it is important to note that the migration of individuals between countries and the increased number of international trips over the years, along with urban developments that have culminated in the megacities spread across the continent, have contributed to the increased and maintained relevance of dengue when it comes to public health in this region [81]. This migratory movement and increased air travel have favored the movement of the dengue virus from endemic regions to disease-free regions, especially due to the arrival of people during the incubation period of the disease, posing a risk of infection of local mosquitoes. An example of this situation occurred on a Chilean island, where dengue resurfaced after years of no reported cases. As a result, dengue has become a serious human health problem in these countries, impacting both health and the economy.

### 4.1. Epidemiologic Issues of Dengue in Latin America

Historically, between the 1950s and 1960s, the Americas were presented as an area free of dengue contamination due to successful vector control campaigns, culminating with the suppression of *Ae. aegypti* [82]. However, during the early and mid-1970s, outbreaks associated with DENV-2 and DENV-3 were reported in Colombia and the Caribbean region. By the end of that decade, DENV-1 had spread to South America, Central America, and Mexico, where more than 702,000 dengue cases were reported from 1977 to 1980 [83]. After that, there was the cessation of vector control campaigns associated with the acceleration of disorderly urbanization, accompanied by management problems in several American cities that favored the recrudescence of the return and movement of the mosquito that transmits dengue. This caused the *Ae. aegypti* mosquito to circulate again in the tropical areas of Central and South America, increasing the incidence of dengue and dengue hemorrhagic fever, with almost 3 million cases officially reported [82].

For example, in Mexico, it has been reported that dengue is responsible for about 140,000 new cases of symptomatic infection each year. In 2019, the country showed the simultaneous circulation of all four dengue serotypes; cases also occurred in the city of Guerrero in Mexico, which is located in the southwest of the country, with an incidence of 24.9 cases per 100,000 inhabitants [84]. It was also reported that there was proof of several cases in Guerrero in 2020, with an incidence of 13.4 cases per 100,000 inhabitants. The concomitant movement of all serotypes was recorded in the cities of Chiapas and Veracruz in 2019, and at least in Nuevo Leon, Tabasco, and Veracruz in 2020 [82,84].

In Central American countries, such as Guatemala and Barbados, dengue is considered endemic and has burdened health systems [85]. In Guatemala, the largest dengue outbreak occurred in 2010, with serotype DENV-2 alone resulting in the notification of 1080 cases [86]. In Barbados, 3994 cases were already reported between 2008 and 2016, and different serotypes circulated during this period, with an emphasis on DENV-1 and DENV-2 [87]. In French Guiana (located in South America), an epidemiological surveillance system capable of collecting data from various sources (e.g., hospitals, health centers, and laboratories) has contributed to monitoring dengue patterns throughout the country. This tool has interesting applicability for detecting outbreaks and providing real-time information to local sanitary authorities [88,89]. Within the epidemiological context, in the period between 2008 and 2013, 1356 dengue cases required hospitalization, 68% of which were classified as dengue with a warning sign [90].

Regarding the end of the second decade of the 21st century, an analysis published by Chen et al. [91] showed that there was a reduction in dengue incidence between the years 2019 and 2020 in Latin America, with the latter year marked by the COVID-19 pandemic. However, experts believe that the pandemic masked the reporting of actual cases of arboviruses in the region, including dengue, especially since, in these localities, one of the main measures to combat the vector is the visits of health agents to the homes of the inhabitants [92]. During the pandemic period, nonpharmaceutical interventions that were characterized by the adoption of sanitary barriers, such as the determination of lockdowns and restriction of mobility, became popular and were widely adopted by government authorities. It is believed that these measures may have interfered with the maintenance of health agents’ activities, such as the identification of suspected cases and referral to health units, which may be related to the lower epidemiological rates of the disease in the period [92].

Another important issue is that one of the main strategies to fight dengue fever depends on community engagement, especially when reducing entomological indexes (possible breeding sites of the mosquito) and other educational measures [93]. This strategy is based on the participation of the local community in the fight against dengue, which requires the population to know, even if only superficially, about different aspects of the disease. The study by Sarti et al. [94] showed that in Colombia and Mexico, more than 65% of the populations evaluated were aware of dengue. However, 70% of the population reported that local governments should invest more in the prevention of the disease. These data strengthen the idea that in Latin America, dengue issues are subject not only to those involving global aspects, such as climate change, but also political and social issues [95].

### 4.2. Economic Aspects of Dengue in Latin America

The economic costs of managing dengue in the Americas are extremely important for managers’ decision-making: planning in countries with scarce resources and the absence of a good quality notification and information system leads to erroneous decisions and expenditure of economic resources that do not mitigate or reduce the damage caused by the disease [96]. According to Laserna et al. [85], the average annual cost of dengue in Latin America was around USD 3 billion. It is noteworthy that, of this total amount, 70% of the direct costs were directed to cases of hospitalized patients. However, it is important to note that the social costs of dengue were significant, since the indirect costs (e.g., productivity losses related to morbidity) could represent up to 80% of the total cost [85]. The study by Shepard et al. [66] showed that in Latin America and the Caribbean, dengue costs were in the range of USD 1.73 billion per year, which is a lower figure than the study published by the same group in 2010, i.e., USD 2.15 billion [82]. Despite this reduction, the study also showed that dengue costs in this region were higher than in other locations, such as Southeast Asia, East Asia, and Oceania or North Africa and the Middle East [66]. An important point was raised by Tiga et al. [97], who reported that economic costs might increase by at least 13% in the case of treating patients with persistent dengue symptoms in Mexico. In this country specifically, the economic burden of dengue is about USD 170 million dollars each year just to control the disease [82]. As in Mexico, in Colombia, the cost of vector control strategies also represents a major portion of the annual burden of dengue, with at least 64% of the total financial cost [74]. However, as shown in Table 1, Brazil is the Latin American country with the highest cost of the disease.

## 5. Dengue in Brazil

Over the last decades, the prominence of Brazil when it comes to the number of cases of the disease in Latin America has been observed. For example, in 2021, PAHO notified more than 1 million cases in the Americas, with the majority of these occurring in Brazil (>76%) [80]. This demonstrates the relevance of the rate of the disease in the country, which proves the need for planning, increased investments, and efforts by competent authorities [98].

In general, Brazil is a country of continental dimensions, with climatic and socioenvironmental variations, and territorial diversity, which mostly favor the development of dengue [99]. Different factors are directly related to this trend, especially the conditions that favor the dissemination of the vector, such as a lack of basic sanitation, inadequate disposal of biological waste, unplanned urban growth, as well as population growth and urban mobility, which are carried out in a disorganized manner [100]. More detailed studies related to the epidemiological chain are necessary, since they can provide more effective responses for dengue intervention and control, especially in the face of conditions in the country that favor the high incidence of the disease [101]. Thus, the socioeconomic impact on the local population can be reduced [19].

### 5.1. Epidemiologic Issues: Focus in Brazil

**Figure 3:**
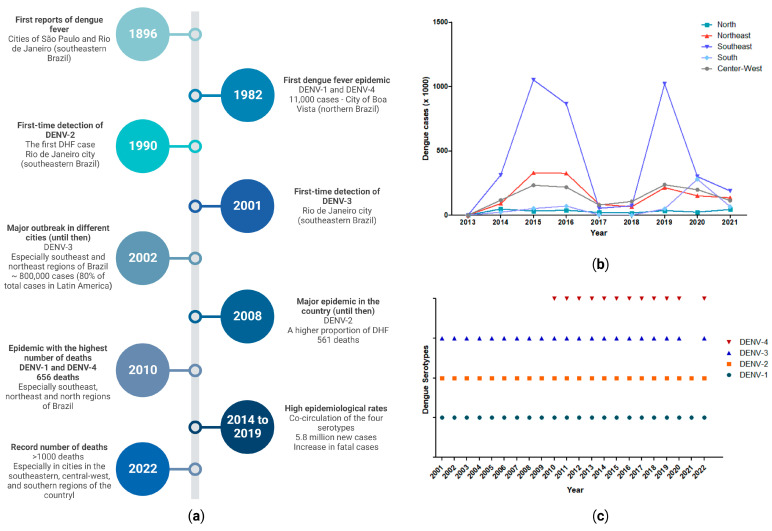
Data on dengue in Brazil: (**a**) timeline with key milestones; (**b**) rate of new dengue cases in the country by region (between 2013 and 2021) (data source: DATASUS [104]); and (**c**) the dengue virus serotypes circulating in the country by year (between 2001 and 2022) (data source: PAHO/WHO [105]). Created in BioRender.com (accessed on 10 March 2023).

Epidemiological data show that dengue is a secular disease in Brazil, since information dating back to the 19th century indicates that dengue epidemics occurred in the states of São Paulo and Rio de Janeiro in 1846 and 1853, respectively; however, the first scientific citations date back to 1916 and 1923 in the cities of São Paulo and Niteroi, both in Southeastern Brazil (Figure 3a) [8]. Evidence indicates that the dengue virus circulated through the Brazilian Amazon, but there is only proof of an epidemic in the city of Boa Vista in the state of Rondônia in 1982, due to the circulation of DENV-1 and DENV-4 (the first time these circulated in the country) [102]. Decades later, DENV-4 returned to Northern Brazil in 2010, approximately 30 years later, being detected in the state of Roraima [103]. In general, in the early 1990s, dengue outbreaks were limited to the southeast and northeast, specifically in cities in the states of Rio de Janeiro, Ceará, Alagoas, and Pernambuco, as well as notifications in the central west, with cases restricted to Mato Grosso and Mato Grosso do Sul [8]. However, the viral movement of DENV-1 and DENV-2 in the subsequent years followed the flow of the *Ae. aegypti* mosquito, showing rapid circulation and reaching other regions of the country, such as the central west, which had the highest incidence rate in the country, with about 450 per 100,000 inhabitants [101].

Furthermore, the stabilization of dengue cases was noted in some Brazilian municipalities. However, the disease remains at a very high level, as is the case in municipalities in the states of Rio de Janeiro, Goiás, and Rio Grande do Norte [101,106]. It is noted that dengue epidemics in Brazil have shown a cyclical pattern, where interepidemic periods have a maximum duration of 4 years, usually with a change in the predominant serotypes [107]. For example, between 2014 and 2019, more than 5.8 million cases of dengue were reported in Brazil, with incidences close to 1 million cases per year (Figure 3b). This trend can be explained by two events: the increase in rainfall in this period, as well as the introduction of a new serotype of the disease, DENV-2, which was barely circulating in the country before 2018 [108]. An interesting analysis that helps understand the dynamics of dengue in Brazil was published by Guzzetta et al. [109], who demonstrated that dengue transmission could occur due to human distance movements of <1 km, with this being a behavior that is observed in a nonendemic metropolis regarding the disease in the country.

An important point comes from data from the Pan American Health Organization (PAHO) that show that Brazil is one of the countries in Latin America with all four dengue virus serotypes circulating at the same time in the last 10 years (2010–2020) (Figure 3c) [105]. The co-circulation of serotypes can lead to the occurrence of simultaneous infections, resulting in genetic recombination events and, consequently, the emergence of more virulent isolates [110]. This may pose challenges related to the management of infected patients. Additionally, after two years of a lower incidence of dengue cases (2017 and 2018) (Figure 3b), it was unclear to the experts which strain was responsible for the outbreak that occurred in 2019, since four DENV lineages were already circulating in the country and causing broad community immunity. The study by Brito et al. [111] pointed out that the high dengue rates that occurred in 2019 were caused by DENV-1 and DENV-2 strains that had already been circulating in Brazil for at least five years. With this, the authors demonstrated that an outbreak is not always caused by the introduction of a new strain, as well as the ability of DENV to survive periods of low transmission, reinforcing the need to establish active genomic surveillance. The above points demonstrate the complexity of viral dynamics and, consequently, the fight against the disease in the country.

### 5.2. Economic Burden: Focus in Brazil

The economic part within this context should also be emphasized, wherein most of the costs of dengue in Brazil are associated with prevention and vector control. As evidenced in Table 1, the economic costs of dengue fever in Brazil are the highest when compared to other countries in Latin America and even Asia. The study by Martelli et al. [112] reported that the costs of dengue fever in Brazil between 2012 and 2013, during which the country was experiencing an epidemic, ranged from >USD 468 million (only considering the reported cases) to >USD 1.2 billion (when considering under-reporting). When looking at the regions of the country, it is noted that the States in the southeast and northeast have the highest dengue costs, representing 21% and 48% of national costs, respectively, which may be related to their higher rates of hospitalization when compared to other locations [113]. The overall average cost per dengue case was USD 531.63 [66]. However, it is important to note that this amount can vary by region, age, clinical management, and when considering the public versus the private sector [112]. For example, the costs of living in Brazil are different depending on the region, which may imply price variations in the acquisition process of healthcare inputs. In addition, it is noted that the costs related to medical staff (contract values) are higher in the private sector than in the public sector.

When analyzing the economic impact of using dengue vaccination in the country, we considered the cost-effectiveness of vaccination (producing good results without costing a lot of money). Brazil has an approximate value of USD 237 to 534 per dose [114], while in Colombia, this value is USD 39.03 [115]. Godói et al. [116] demonstrated that the cost per dose of the CYD-TDV vaccine in the country could reach USD 33.61, a higher value than that of a dose of the trivalent influenza vaccine (used in annual mass immunization campaigns) (~USD 2.90) [117]. Despite the cost and limitations of its efficacy, Shim [118] reported that dengue vaccination would be cost-effective in Brazil.

### 5.3. Disease Control: Focus in Brazil

As is the case of other countries, dengue control in Brazil is directed at the mosquito that transmits the disease [119]. Even with the CYD-TDV vaccine being approved in the country since 2015, due to restrictions of its indication, public policies have been directed to combat the *Aedes* species [120]. Among the efforts observed, one can notice the fight against the breeding sites of the vector, an action performed by professionals called endemic disease agents or health agents [121]. However, in the recent years, there has been an increasing decline in the number of these professionals hired, whether at the federal, state, or municipal level, resulting in the population having less access to this service. It is also noteworthy that the lifestyle of modern society also favors the dissemination of these vectors, either by the high rates of violence, making communities resistant to health agents visiting their homes to support the elimination of the breeding sites of the transmitting mosquito, as well as the habit of disposing of packaging and items in an improper way, which, for the most part, have a short life span, ending up as breeding sites [15]. Thus, allied to this issue is the disorderly urbanization observed in different Brazilian cities, which results in the improper disposal of garbage, such as tires and disposable bottles, which are dumped in vacant lots, as well as other breeding spaces for the mosquitos, such as old iron yards, which provide the accumulation of water and thus the multiplication of vectors [122]. An example of this contextualization occurred in 2008 during a dengue epidemic in the city of Rio de Janeiro, where high demographic concentrations, uncontrolled urban growth, and health determinants that were below those acceptable with regard to public health were crucial to the growth of dengue incidence in individuals of <15 years of age, including the most severe forms of the disease [123]. It is highlighted that dengue is more common in individuals aged 20–39 years in the country [107].

Additionally, like other places in the world, Brazil has also adopted measures, such as the use of insecticides (especially organophosphates and pyrethroids) against sites with a high population density, as well as the use of genetically modified mosquitoes to control vector reproduction [122,124]. However, the resistance of *Ae. aegypti* populations to these chemical compounds has already been reported in different cities across the country, with further evidence that the process of resistance reduction can be difficult and slow, requiring at least 7 years for a possible reversal [125]. One point that requires attention is that, even with this evidence, local governments have used the same strategy to combat other *Ae*. *aegypti*-transmitted viruses, indicating that there might be an even higher rate of resistant populations [119].

## 6. Considerations and Perspectives for Dengue: Focus on Epidemiology

As presented in the previous topics, the fight against dengue presents several challenges that involve everything from the development of effective therapies, to the need for community engagement to combat the vector of DENV transmission. From the epidemiological point of view, the resurgence of dengue has become a challenge for public health not only in endemic regions (especially in Brazil and other Latin American countries), but also in places that did not present cases of the disease. Based on an analysis of the challenges inherent in combating this disease, different approaches have been proposed to support the elucidation of the impacts of dengue, including within an epidemiological context.

### 6.1. General Perspectives and Challenges Regarding Dengue

Dengue is one of the most important arthropod-borne viral diseases and is found in more than 100 countries [126]. An interesting point is that, in 1950, only nine countries had reported cases of dengue fever on their territory, which leads one to reflect on how quickly this disease spread in a short period of time when compared to the spread of other diseases worldwide [127]. The main reason responsible for the resurgence of dengue is that the vector leads to the dissemination of the virus, which is a direct consequence of the deforestation and destruction of the natural habitat of these insects [1]. Allied to this situation, over the years, we have observed the adoption of mistaken public policies by the authorities, whether in the social field or in the field of prevention and health promotion, which leads countries, especially the poorest ones, to make decisions that are crucial for the return of dengue [128]. Thus, dengue has been indicated as the disease with the greatest potential risk to the world population and is associated with different challenges. Within this perspective, it is important to highlight that, according to the WHO, 60% of the notifications that are registered come from the Americas, with Canada being the only exception in this region [81].

The renunciation of vector control policies that are associated with demographic displacement that causes the migration and establishment of these vectors in other locations, the consumption habits of modern society that cause a substantial increase in the accumulation of recyclable or nonrecyclable waste, and the lack of perception that the disease can bring serious risk to health and life are responsible for the aggravation of this situation on the American continent and its countries [129]. In order to achieve results that are compatible with the health needs of the population, this context can only begin to change when policies of promotion, prevention, education, and the participation of society associated with combating the vector and the disease through epidemiological surveillance are properly implemented [130]. Brazil is a country located in South America that portrays this reality. Most of the new dengue cases in Latin America are due to environmental, social, and economic issues, and dengue epidemics in Brazil have presented a cyclical pattern. Even with different control strategies being established by the responsible authorities, the disease is still a serious obstacle to the country’s public health [131].

Just like Brazil, other countries in the Americas are marked by poverty or unequal income distribution, generating concerns for public health. In a specific way, the incidence of dengue in these countries has become a reason for extreme apprehension in society, and particularly for health managers, due to the difficulties presented in combating and controlling the disease, as well as the need to increase the supply of services to deal with the contamination of the most severe form of the disease, especially dengue hemorrhagic fever [8]. Importantly, the WHO classifies dengue as a neglected tropical disease (NTD), which means that it is a disease with little space on the global health agenda, as well as receiving little financial input from investors [132]. Horstick et al. [133] reported an intriguing analysis of this classification, where they showed that dengue fever could not fit into part of the WHO requirements for an NTD. For example, even though most of the countries affected by dengue have limited economic resources, the population of these places with greater purchasing power can also be infected [133]. Additionally, investments in the research and development of new therapies for dengue have increased exponentially in the recent years [134]. However, even if these aspects differ when compared to other NTDs, such as malaria and Chagas’ disease [135], it is noted that the need for well-structured surveillance, the difficulty in controlling the vector, and the absence of effective vaccines mean that dengue can still be treated as an NTD [133].

### 6.2. Perspectives on Economic Epidemiology

New study mechanisms and technologies bring subsidies for better health planning. In the field of economics, a new branch called economic epidemiology analyzes how economic tendency affects the spread of pests and their respective pathogens [136]. To this end, this approach proposes to determine a relationship between preventive standards and disease prevalence, as well as the relationship between economic causes and the epidemiological consequences of the number and type of contact people make [137]. Specifically, economic epidemiology considers the possibility that the demand for self-protection against a particular disease is sensitive to the prevalence of the disease (proportion of the population affected by a specific disease in a given period), i.e., the more prevention (or protection), the less disease, while the more disease, the lower the rates of prevention [138]. This cyclical pattern allows an analysis of the behavior that changes in response to new incentives created by the growth of a disease, as well as the effects of these changes on public health and economic measures [139].

From the perspective of economic epidemiology, in Brazil, there is the National Program of Primary Care, which demonstrates that, based on the care and empowerment of the individual, through knowledge, skills, competencies, and confidence, the development of necessary requirements for management and decision-making regarding their own health occurs [140]. In this way, it is believed that positive results occur indirectly within the public health sphere. This behavior becomes extremely important in the fight against dengue, since the population’s engagement can result in reducing the spread of the vector and, consequently, the occurrence of the disease. Thus, by bringing this issue into the light of economic epidemiology, the adaptation of health behaviors of populations in response to the high prevalence of the disease can culminate in more effective public policies, since it would be based on protective practices [141].

### 6.3. Perspectives on Technological Advancement and Support of Epidemiological Elucidation

Another important issue is technological advances and their association with epidemiology and public health. For example, data analysis mechanisms (through computer tools) in projection and prediction studies have been used systematically, aiming to maximize resources and optimize results in action planning, especially in countries with limited financial resources, such as those in Latin America [142]. The study by Cabrera et al. [95] demonstrated that machine learning techniques could be successfully employed in dengue prediction models, being an important tool for Latin countries. In Brazil, a program called “R” has been used within the public health service (known as SUS), allowing for the better analysis of statistical data related to SUS and reducing the work in the systematization of data, generating more information in less time [143]. This has enabled better planning with fewer possibilities of errors and unnecessary financial costs in health management. These tools, such as the “R” package, are responsible for generating health indicators that will allow managers to monitor the behavior of their health policies, identifying this during the course of the same trends and priority regions, where resources can be better distributed and allocated, bringing the best employability of resources with better results to countries with fewer resources [144].

### 6.4. Perspective on Dengue and Wastewater-Based Epidemiology

Finally, it is important to highlight an epidemiological tool with broad prospects for future use: wastewater-based epidemiology (WBE). WBE is based on the possible detection of biomarkers in sewage, including viral genetic material [145]. In general, the application of WBE is considered to be a cheaper and more efficient alternative for tracking infectious agents in communities, which makes its use feasible in countries with limited financial resources and a wide range of circulating pathogens, such as Brazil and other regions of Latin America [146]. Moreover, this tool is considered a complementary alternative to traditional surveillance strategies, since it allows for the detection of a virus in human excreta samples from symptomatic or non-symptomatic individuals [147], which is an important factor for dengue, with a broad spectrum of symptoms and with many of them similar to other diseases. This methodology can also support the adoption of public policies, since the detection of a higher viral concentration can indicate that there is a large number of infected individuals in a particular region, and there may be the need to promote programs to control the transmission vector, such as actions focused on education and social mobilization. The decrease in the under-reporting rate can be a consequence of this initiative, as well as the strengthening of the fight against the transmitting mosquito. Currently, the application of WBE for dengue still has gaps in knowledge to be answered [148]; however, the expectation is that, in the future, this methodology will contribute significantly to the fight against dengue [149].

## 7. Conclusions

This review allowed us to ascertain that dengue is still an arbovirus with a high incidence rate, especially in poorer countries. This imposes large financial burdens on the coffers of these countries, and is a great inconvenience for their populations and respective economies. We understand that dengue has ceased to be an endemic disease, becoming of great importance for social policies, with enormous socioeconomic impacts. The literature has also pointed out a need to formulate policies horizontally, in order for them to be successful, taking into account the epidemiological data collected and the real needs of the population in order to allow for the mobilization of whole societies in a joint effort to eradicate this disease.

## Figures and Tables

**Table 1:** Overview of the epidemiology of dengue fever in the world, including the number of new cases in 2022 [57] and the level of risk of infection [58].

Country	New Cases Reported in 2022	Level of Infection Risk
Asia		
Afghanistan	1266	Sporadic/Uncertain
Bangladesh	82,743	Frequent/Continuous
Cambodia	12,591	Frequent/Continuous
China	537	Risk varies based on region
India	110,473	Risk varies based on region
Indonesia	125,888	Frequent/Continuous
Malaysia	64,078	Frequent/Continuous
Nepal	54,784	Frequent/Continuous
Pakistan	78,554	Risk varies based on region
Philippines	220,705	Frequent/Continuous
Singapore	31,883	Sporadic/Uncertain
Thailand	33,489	Frequent/Continuous
Vietnam	367,729	Frequent/Continuous
Africa		
Kenya	34	Frequent/Continuous
São Tomé and Príncipe	1161	Sporadic/Uncertain
Somalia	5350	Frequent/Continuous
Sudan	4800	Frequent/Continuous
American		
Brazil	2,363,490	Frequent/Continuous
Colombia	69,497	Frequent/Continuous
Mexico	59,918	Risk varies based on region
Nicaragua	97,541	Frequent/Continuous
Peru	72,851	Risk varies based on region
Australia and the Pacific		
Australia	407	Frequent/Continuous
Fiji	1960	Frequent/Continuous
Vanuatu	148	Sporadic/Uncertain
Europe		
France	272	Sporadic/Uncertain

**Table 2:** Dengue-related costs in different countries across five continents.

Region	Country	Dengue-Related Costs (in USD)	Reference
Asia	Vietnam	The average cost for the ICU per inpatient is between 64.4 and 4250	[67]
	China	Total economic costs in 2019: ~460 million	[68]
	Indonesia	The aggregate cost of illness in 2010: >300 million The average cost per capita: >1.3	[69]
	Thailand	The aggregate cost of illness in 2010: >290 million The average cost per capita: >4.34	
	Singapore	The aggregate cost of illness in 2010: >67 million The average cost per capita: >12.65	
	Cambodia	The aggregate cost of illness in 2010: >16 million The average cost per capita: >0.11	
Africa	Burkina Faso	The average cost of illness per inpatient: 26 The average economic costs per episode, considering outpatients: 13	[70]
	Kenya	The average economic costs per episode considering outpatients: 33	
America	El Salvador	The aggregate annual cost of illness: 1.7 million	[71]
	Venezuela	The aggregate annual cost of illness: 10.2 million	
	Guatemala	The aggregate annual cost of illness: 1.2 million	
	Panama	The aggregate annual cost of illness: 0.9 million	
	Cuba	The average cost per hospitalized case: >296	[72]
	Puerto Rico	The average cost per capita: 31.52	[73]
	Honduras	The average cost per capita: 2.12	
	Colombia	The total financial cost of the illness: 131.7 million	[74]
	Brazil	The annual aggregated cost of illness: 1014.3 million The average cost per dengue case: 531.63	[66]
Oceania	Australia	Total annual cost of illness: >2.7 million	[75]
Europe	Italy	The average cost per dengue case: ~290	[76]

## Data Availability

Data are contained within the article.
